# I am Once Again Asking for Your Attention: A Replication of Feature-Based Attention Modulations of Binding Effects with Picture Stimuli

**DOI:** 10.5334/joc.432

**Published:** 2025-02-05

**Authors:** Tarini Singh, Lars-Michael Schöpper, Christian Frings

**Affiliations:** 1 University of Trier, DE

**Keywords:** Action and perception, Attention, Emotion and cognition

## Abstract

Action control theories assume that stimulus and response features are integrated or bound into short term episodic traces. A repetition of any of these features results in a retrieval of the entire episodic trace, and can thus facilitate or interfere with future actions. Along with stimuli features, features of the response and any other irrelevant stimuli that are present, are also integrated into such traces and can influence future actions. Using word stimuli, Singh et al. (2018) observed that such so-called binding effects are larger for attended features relative to unattended features. This was the case even for features generally believed to be automatically processed, like valence. Since previous research has shown differences in the processing of word and picture stimuli, it is questionable whether the attentional modulations in the above study would extend to picture stimuli. In order to examine this question, Experiment 1 replicated the design of Singh et al. (2018) but used picture instead of word stimuli. In order to directly compare word and picture stimuli, the data of Singh et al (2018) were re-analysed together with the data of the present study. In Experiment 2, the alternative hypothesis, that the effects were driven by the encoding of stimulus contingencies, was tested. Taken together, the findings of the present study replicate those of Singh et al. (2018), indicating that even with picture stimuli, valence related binding effects are modulated by attention allocation.

## Introduction

In order to efficiently plan and execute actions, information from different sources must be integrated, for instance, perceptual features or different response features of the planned or executed action that may be processed in different regions of the brain (e.g., Treisman, 1999) and may need to be integrated before the execution of an action. Stimulus and response features of a single event are integrated and stored together in temporary episodic traces called *event files* (Hommel et al., 2001). If any part of the information contained within them is re-encountered, the entire event file, is retrieved – stimulus-response binding effects emerge (e.g., Frings et al., 2020; Henson et al., 2014; Hommel et al., 2001). While more recent action control frameworks (e.g., BRAC, Frings et al., 2020) differentiate between a binding (or integration) process and a retrieval process, both of which can be independently manipulated, for the present purposes we refer to the effect as a whole. Therefore, we use the term *binding effect(s)* to refer to the effect as a whole. Re-encountering information that is contained in an active event file can influence responding. The retrieved event file only interferes with current actions if the information is partially repeated. If all of the information is repeated, that is, completely overlaps with the event file, there is a perfect match between the current information and the information in the retrieved event file. If none of the information is re-encountered, nothing will be retrieved in the first place. If, however, only some, but not all of the information is re-encountered, then the repeated information leads to a retrieval of the event file. In this case, the current information does not completely match the information in the retrieved event file. This mis-match causes interference with actions, that is, slower responding or more errors, called *partial repetition costs* (Hommel, 1998).

Along with relevant information, event files can also contain information irrelevant to the current task, for instance information about irrelevant stimuli that occur simultaneously or in close temporal contiguity with the relevant stimuli, or information about irrelevant features of the target stimulus (e.g., Frings et al., 2007, 2020), such as colour (Schöpper & Frings, 2022; Singh & Frings, 2018), shapes (Singh & Frings, 2018; as frames, see Schöpper et al., 2022), location (Schöpper et al., 2020; Singh & Frings, 2018), flanking letters (Frings et al., 2007). Such irrelevant information can also influence future responses – *distractor-response binding* (Frings et al, 2007). Distractor-response binding is a specific case of stimulus-response binding; the integration or binding and retrieval of irrelevant stimulus information and response features. Distracting information can either facilitate or interfere with responding depending on whether the response information remains the same or changes, that is, the outcome of repeating a distractor depends on whether the response information between two occurrences of a distractor remains constant or changes. If both distractor and response remain the same between two occurrences of the same distractor then the repeated distractor will facilitate responding. The repeated distractor automatically retrieves the previous event file, which contains response information that matches the currently required response information (since the response has not changed). If, however, the response between two occurrences of a distractor changes (while the distractor repeats), then response interference (longer reaction times and/or higher error rates) is observed. The repeated distractor automatically retrieves the previous event file, however, the response information contained in the event file that is retrieved by the repeated distractor no longer matches the currently required response information (since the response has changed) and thus interferes with response selection (e.g., Frings et al., 2007, 2020). The magnitude of the distractor-response binding effect, which is indicated by a significant interaction between the distractor and response, can be quantified by first computing the difference between distractor repetitions and distractor changes when the response repeats and between distractor repetitions and distractor changes when the response changes, and then computing the difference between these differences.

While such integration and retrieval effects are relatively automatic (Frings et al, 2020; Hommel et al., 2001), they are still susceptible to modulation via attentional processes (e.g., Moeller & Frings, 2014; Singh et al., 2018; however, see Schöpper, Küpper, & Frings, 2023, for an absence of modulation by attentional biases). For instance, Singh et al. (2018) observed that when two distractors are present, distractor-response binding effects are stronger for the distractor which is currently attended, compared to the unattended distractor. This is the case even when the distractor is only relevant for a different task. In their study, Singh et al. (2018) instructed participants to respond to the colour of a word, while the meaning of the word was irrelevant for the speeded reaction time task, in which binding effects for each feature were measured. For the attentional manipulation, a secondary task was introduced in which participants were instructed to report either the valence or word type of the target word. A feature was considered an *attended* feature if it had to be reported in the secondary task, that is, was relevant to the secondary task and *unattended* if it did not have to be reported in the secondary task, that is, was irrelevant to the secondary task. Interestingly, this modulation was observed even for features like valence (Singh et al., 2018), which has generally thought to be processed independently of attentional resource allocation (e.g., Fazio et al., 1986; Kissler et al., 2009; Moors et al., 2005; Pecchinenda & Heil, 2007).

### Valence processing and attention

Processing of emotional valence has long been considered to be automatic. In their seminal paper, Fazio et al. (1986) interpreted their findings in an affective priming task as evidence that emotional valence processing is spontaneous, and inescapable. In an affective priming task, generally, a prime stimulus that is affectively congruent or incongruent with the target stimulus is presented. Typically, faster responses (and fewer errors) are observed for congruent relative to incongruent prime-target combinations. Fazio et al. (1986) presented participants with individually pre-tested positive and negative attitude objects as primes in a priming task with affective adjectives as targets (they additionally differentiated between strong and weak primes). Primes were presented briefly for 200 ms followed by an interval of 100 ms before the target was presented. They observed that briefly presenting the positive and negative attitude object primes resulted in significant priming effects. They took this as evidence that positive and negative attitudes can be automatically activated by the stimulus. A number of studies have since observed evidence that affective or emotional valence processing is automatic. For instance, Hermans et al. (1994) extended the design of Fazio et al. (1986) by using different stimulus complexity and a different response modality and also observed significant affective priming effects. Even in tasks in which valence is not part of the primary task, evidence has been observed for the automatic processing of emotional valence. For instance, one study by Hartikainen et al. (2000) observed that even when participants were instructed to attend only to the orientation of a target triangle, affective images presented to the left visual hemifield prior to the target interfered with performance compared to neutral images. In another study, Fischer and Schubert (2008) tested whether affective processing is constrained by limitations on central resources. They used a psychological refractory period (PRP) paradigm, in which two tasks are presented at different stimulus onset asynchronies (SOA) ranging from short to long intervals. Generally, reaction times for the second task are longer at shorter SOAs, a finding attributed to the capacity limitation at the response selection stage. In their study, Fischer and Schubert (2008) observed that affective processing was able to bypass the central bottleneck during dual task performance. An EEG study looking at event related potentials (Kissler et al., 2009) observed significant early posterior negativity (EPN), that is, a more negative signal for emotional compared to neutral stimuli, over the occipital area between 150–300 ms after stimulus onset, a component believed to reflect automatic processing of emotional stimuli relative to neural stimuli (Schupp et al., 2006), when viewing emotional words even when valence was not relevant to the task.

On the other hand, a growing body of evidence suggests that valence processing is not completely automatic and is indeed subject to attentional resources. For instance, Pessoa et al. (2002b) presented participants with fearful or neutral faces at the centre of the screen along with a horizontal or vertical bar on either side of the face. In attended trials, participants reported the gender of the face and in unattended trials participants reported whether the lines were both of the same orientation. They observed significantly lower activation of the amygdala in the unattended trials, indicating that emotional processing is to some extent subject to attentional resources. In an affective priming study, Spruyt et al. (2009) only observed significant affective priming effects when participants were instructed to categorise the valence of the words but not when participants simply read the words out aloud. Similarly, Singh et al. (2018) observed significant binding effects for valence only when valence was relevant to a secondary task, but not when another feature was relevant to the secondary task. Taken together these findings suggest that while valence or affective processing might be largely automatic it is still subject to constraints on attentional resources.

### Valence processing in word and image stimuli

In addition to the various findings both for and against the argument that valence processing underlies attentional constraints, there is also evidence that valence processing differs, at least to some extent, for word and image stimuli. Some evidence suggests that emotional words and images are processed in different ways (e.g., Beall & Herbert, 2008; De Houwer & Hermans, 1994; Feng et al., 2021; Hinojosa et al., 2009).

A number of studies have compared the processing of emotional word stimuli and emotional image stimuli and have found differences both in behavioural effects as well as in the neural underpinnings (e.g., Beall & Herbert, 2008; Bayer & Schacht, 2014; De Houwer & Hermans, 1994; Feng et al., 2021; Flaisch et al., 2015; Kensinger & Schacter, 2006). For instance, De Houwer and Hermans (1994) presented participants with picture-word stimuli combinations of positively and negatively evaluated animals and instructed one half of the participants to categorise the valence of the picture and the other half were instructed to categorise the valence of the word. They observed that while picture categorisation was not influenced by the word valence, the categorisation of the word stimuli were significantly influenced by the picture stimuli. Word categorisation was significantly slower with an incongruent picture than with a congruent picture. However, in a further experiment, in which the words and pictures were named rather than categorised, interference effects were observed in the picture naming task but not in the word reading task. De Houwer and Hermans (1994) concluded that pictures might have privileged access to affective information. Schacht and Sommer (2009) observed more or less similar scalp topographies of the event related potential (ERP) components elicited by word and picture stimuli, however, ERP components elicited by picture stimuli emerged earlier than for word stimuli. They concluded that similar processing systems are involved for word and picture stimuli, with picture stimuli having a speed advantage over word stimuli. Beall and Herbert (2008) observed that both words and faces cause interference when categorising the valence of the stimuli, however, the interference effect via faces was significantly larger than the interference effect via words. However, in this study the pictorial stimuli were exclusively faces, which are known to be processed differently to other kinds of stimuli (e.g., Grill-Spector et al., 2004; Kanwisher et al., 1999). Additionally, Spruyt et al. (2002) observed that affective priming effects are more reliable with picture stimuli than with word stimuli in tasks in which no valence classification was required. Lastly, Schöpper, Jerusalem, et al. (2023) observed that completely task-irrelevant valence operationalized by fruits and spider images was retrieved by discrimination responses – suggesting that even irrelevant valence can be bound to a response. It is therefore possible that the findings with regards to valence-response binding effects in the Singh et al. (2018) study were driven, at least partly, by the use of words instead of pictures as stimuli.

## Present Study

Taking into account the differences in valence processing for word and picture stimuli, the aim of the present study is to test whether the findings of Singh et al. (2018) with respect to attentional modulation of valence-response binding effects, that is, the binding of the stimulus feature valence and the response, was due to the use of words instead of picture stimuli. Given that differences have been observed in the processing of affective words and images, it is possible that the previous findings were driven by the use of words rather than images. That is, it is possible that the observed attentional modulation of valence related binding effects in Singh et al. (2018) might not be observed when using picture rather than word stimuli. In order to test this possibility, the present study implements the same design as Experiment 1 of Singh et al. (2018) with the exception that instead of word stimuli, picture stimuli were used. Singh et al. (2018) presented participants with words that were either positive or negative and either nouns or adjectives. Participants were instructed to respond only to the colour of the word. Additionally, in one condition, participants were asked to report the valence of the words in 75% of the trials and in another condition, participants were asked to report the lexical category (word type) of the words in 75% of the trials. In both conditions, distractor-response binding effects were measured for valence (i.e., valence-response binding effect) and for word type (i.e., word type-response binding effect). When participants were asked to report the valence of the word in 75% of the trials only a significant valence-response binding effect was observed but no word type-response binding effect was observed. Conversely, when participants were asked to report the lexical category of the word in 75% of the trials, only a significant word type-response binding effect was observed but no valence-response binding effect, indicating that only if a feature receives attention, it is, integrated with the response.

If valence processing is not wholly automatic and does rely on attentional resources, and valence processing of pictures and words do not differ, then the findings of Singh et al. (2018) should be replicated using pictures instead of words as stimuli, that is, valence-response (and picture category-response) binding effects should only be observed when attention is explicitly allocated to valence (or picture category) processing. If, however, valence is indeed somehow special and profits from a prioritised processing, and the previous failure to find valence-response binding effects was due to the use of word stimuli instead of pictures, then using picture stimuli should produce a valence-response binding effect even if attention is allocated away from valence to some other stimulus feature, while picture category-response binding effects should only be observed when category is attended. Specifically, this could be one of at least two different outcomes. Firstly, valence-response binding effects are not modulated by attentional allocation at all, that is, the valence-response binding effects are the same irrespective of whether valence or category is attended. The second possibility is that valence-response binding effects are indeed modulated by attentional allocation, but this is only a modulation of the magnitude of the effect. That is, significant valence-response binding effects would still be observed when valence is not attended, however these effects would be smaller than when valence is attended. The latter would indicate that the attentional modulation of binding effects in action control is not an all-or-nothing phenomenon, but rather depends, at least to some extent, on the intrinsic properties of the stimuli.

Thus, the relevant finding in the present study, is the presence or absence of a valence-response binding effect when valence is not attended, that is, when category is attended. Similarly, as a comparison for a non-affective feature, the relevant finding is the presence or absence of a category-response binding effect when the category is not attended, that is, when valence is attended. In order for participants to attend to one or the other feature, without making those features response relevant, a secondary task was introduced (Singh et al., 2018), in which participants were additionally instructed to report the valence or category of the stimulus on 75% of the trials. Thus, while a feature may be relevant to the secondary task, thus increasing the amount of attention directed towards it, neither feature was relevant to the response in the primary task, in which the binding effects were measured.

## Experiment 1

### Method

#### Participants

Sixty participants from the University of Trier (45 female) participated in the study as part of the course requirement. Participants signed up for the study via an online recruiting system (SONA) or were recruited by the experimenters on campus and were then assigned to a condition. In order to counterbalance the response mapping, each attended condition included two response mapping conditions. Participants were assigned to each of the conditions in an alternating manner. The median age of the participants was 21 years (range 18–33 years). All participants reported normal or corrected-to-normal vision. The sample size was based on Singh et al. (2018), who collected a sample of 60 participants (30 per attended condition). One participant was excluded from the analysis since they had no valid observations in two conditions. Six further participants were excluded from the sample due to being outliers in error rates^1^ leading to a final sample of 53 participants.

#### Design

The present study consisted of a 2(response relation: repetition vs. change) × 2(valence relation: repetition vs. change) × 2(picture category relation: repetition vs. change) × 2(attended feature: valence vs. picture category) design. The first three factors were varied within subjects, while the factor attended feature was varied between subjects.

#### Materials and apparatus

Experiments were run with the E-Prime 3 software (Psychological Software Tools) on a tower PC running Windows 10 with an Intel Core i3 chipset and 16 GB RAM, attached to a 22-inch monitor with a 16:10 format and a resolution of 1680 × 1050 pixels. Responses were given on a standard German language layout QWERTZ keyboard. Stimuli were taken from the Geneva Affective Picture Database (Dan-Glauser & Scherer, 2011) and International Affective Picture Database (Lang et al., 1997) based on valence ratings and picture category. A total of 48 images, 24 aversive, 24 non-aversive, 24 animate objects (humans, animals), and 24 inanimate objects (objects, landscapes, scenes of car accidents). This led to a total of 12 stimuli in each category combination, that is, aversive animate, aversive inanimate, non-aversive animate, and non-aversive inanimate. The image IDs along with the valence ratings are presented in **Appendix B**. The images were presented in black and white on a black background. The stimuli were presented in either a yellow (RGB values: R:228, G:255, B:0) or a green (RGB values: R:144, G:255, B:0) border. The images were 14.34° × 9.32° degrees visual angle and the border was 0.1° visual angle.

#### Procedure

**Figure 1:**
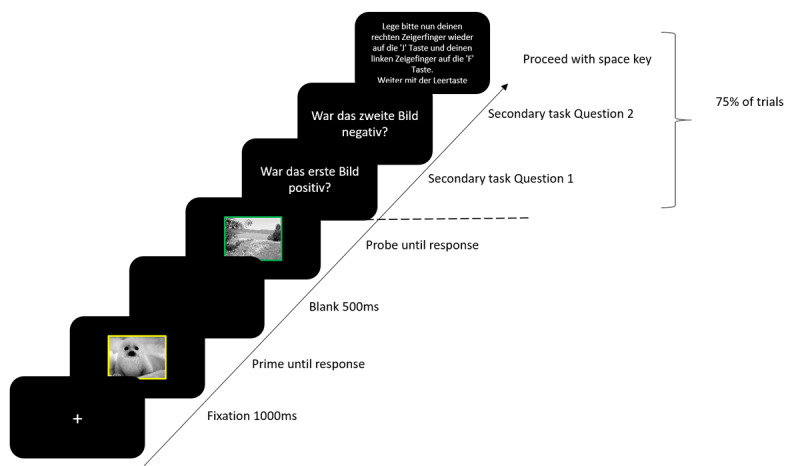
Trial sequence. Secondary task Question 1 translates to “was the first image positive?”. Secondary task Question 2 translates to “was the second image negative?”. Questions could be regarding the valence or the category of the stimulus. For valence, the question could refer to whether the image was positive or negative. For category, the question could refer to whether the image was animate or inanimate. The ‘Proceed with space key’ instruction translates to “Place your right index finger back on the ’J’ key and your left index finger on the ‘F’ key. Proceed with the spacebar.”

Participants were tested in a laboratory with two testing stations. Participants were seated 60 cm from the screen. Participants gave their informed consent before beginning the experiment. Experimental instructions were presented on screen. Participants were instructed to place their left and right index fingers on the F and J keys respectively, and to only respond to the colour of the border of the image as quickly and as accurately as possible. They were also informed that on some trials they would be asked a question about the prime and the probe stimulus which they were to respond to with the 4 and 6 keys on the number pad. Prime and probe stimuli were selected at random from a list of stimuli for that specific condition, with the constraint that stimuli were never repeated from the prime to the probe. Each trial began with a fixation marker for 1000 ms. Next the prime display was presented until a response was registered followed by a blank display for 500 ms. The blank display was followed by the probe display until a response was registered. On 75% of the trials, the probe display was followed by a yes/no question about the valence or category of the prime stimulus followed by a yes/no question about the valence or category of the probe stimulus. After responding to both yes/no questions, participants were instructed to place their left and right index fingers on the F and J keys respectively and to press the space bar to start the next trial (Figure 1). On the 25% of trials in which no question were presented, the next trial began automatically. For one half of the participants the questions always referred to the valence of the stimuli (attended feature valence) and for the other half the questions always referred to the picture category of the stimuli (attended feature picture category). In the valence attended condition, participants were either asked whether the picture was positive or whether it was negative, and in the category attended condition, they were either asked whether the picture was animate or inanimate. The first of the two questions always referred to the prime stimulus (i.e., “was the first picture positive?” or “was the first picture negative?” in the valence attended condition, and “was the first picture animate?” or “was the first picture inanimate?” in the category attended condition). The second question always referred to the probe stimulus (e.g., “was the second picture positive?” or “was the second picture negative?” in the valence attended condition, and “was the second picture animate?” or “was the second picture inanimate?” in the category attended condition). In response repetition (RR) trials, the prime and probe required the same response, in response change (RC) trials, the prime and probe required different responses. In valence repetition (VR) trials the prime and probe stimulus had the same valence, in valence change (VC) trials the prime and probe stimuli had a different valence. It should be noted, that while the valence could be repeated, the stimulus was not repeated from prime to probe. In picture category repetition (CR) trials the prime and probe stimulus were from the same category, in picture category change (CC) trials, the prime and probe stimulus were from different categories. Although picture category could repeat, the stimulus was not repeated from prime to probe. An orthogonal variation of all the factors resulted in a total of eight conditions; response repetition-valence repetition-category repetition (RRVRCR), response repetition-valence repetition-category change (RRVRCC), response repetition-valence change-category repetition (RRVCCR), response repetition-valence change-category change (RRVCCC), response change-valence repetition-category repetition (RCVRCR), response change-valence repetition-category change (RCVRCC), response change-valence change-category repetition (RCVCCR), response change-valence change-category change (RCVCCC). The test block consisted of 32 repetitions of each trial type, resulting in 256 trials in total. Before the test block, the participants worked through a practice block consisting of 32 randomly selected trials. In the practice block, participants received feedback after every response for both tasks, in the test block participants received feedback only after erroneous responses for both tasks. Practice block trials were excluded from all analyses.

Separate binding effects for each distractor were computed as follows; for valence-response binding ((RRVCCR + RRVCCC)/2 – (RRVRCR + RRVRCC)/2) – ((RCVCCR + RCVCCC)/2 – (RCVRCR + RCVRCC)/2)). For picture category-response binding ((RRVRCC + RRVCCC)/2 – (RRVRCR + RRVCCR)/2) – ((RCVRCC + RCVCCC)/2 – (RCVRCR + RCVCCR)/2)).

### Results

#### Reaction Times

RTs were analysed in a 2(response relation: repetition vs. change) × 2(valence relation: repetition vs. change) × 2(picture category relation: repetition vs. change) × 2(attended feature: valence vs. picture category) mixed ANOVA (ez package, Version 4.4-0, Lawrence, M.A., 2016; R Version 4.3.2, R Core Team, 2023) with the first three factors as repeated measures factors and the last factor as a between subject factor. Only probe RTs in trials with correct responses to both the prime and the probe were considered. RTs shorter than 200 ms and longer than 1.5 times the IQR above the third quartile of the individual participants’ distribution (Tukey, 1977) were excluded from the analysis. This led to an exclusion of a total of 15.25% of the trials (5.43% due to being RT outliers, 0.09% due to RTs shorter than 200 ms, 9.74% due to errors in either the prime, the probe, or both). Mean RTs along with SD are presented in Table 1 for picture stimuli, along with the RTs from Singh et al. (2018) with word stimuli. Only the hypothesis relevant results relating to the binding effects are presented here, the full results of the analysis are presented in Table 2.

**Table 1:** Mean RTs and SD in ms for picture stimuli and word stimuli from Singh et al. (2018).


		VR		VC	
	
		CR	CC	CR	CC
	
		MEAN (SD)	MEAN (SD)	MEAN (SD)	MEAN (SD)

Valence Attended					

RR	Picture	833 (219)	824 (198)	898 (266)	907 (272)

	Word	582 (102)	580 (116)	619 9(5)	628 (117)

RC	Picture	912 (248)	923 (268)	875 (240)	894 (262)

	Word	668 (118)	673 (122)	658 (125)	658 (132)

Category Attended					

RR	Picture	786 (207)	839 (249)	801 (231)	827 (230)

	Word	537 (66)	552 (67)	540 (55)	559 (70)

RC	Picture	817 (180)	824 (180)	820 (197)	819 (212)

	Word	592 (57)	592 (64)	588 (66)	584 (44)


VR/VC = Valence repetition and valence change, CR/CC = Category repetition and category change, RR/RC = response repetition and response change.

**Table 2:** Full analysis of RTs in Experiment 1.


EFFECT	DFs	*F*	*p*	η_p_^2^

Attended Feature (A)	1, 51	1.17	.284	.02

Response Relation (R)	1, 51	5.99	.018	.11

Valence Relation (V)	1, 51	3.95	.052	.07

Category Relation (C)	1, 51	8.96	.004	.15

A × R	1, 51	2.81	.100	.05

A × V	1, 51	3.89	.054	.07

A × C	1, 51	2.09	.155	.04

R × V	1, 51	20.89	<.001	.29

R × C	1, 51	0.94	.338	.02

V × C	1, 51	0.06	.802	.00

A × R × V	1, 51	19.29	<.001	.27

A × R × C	1, 51	5.32	.025	.09

A × V × C	1, 51	3.53	.066	.06

R × V × C	1, 51	0.07	.797	.00

A × R × V × C	1, 51	0.54	.464	.01


**Figure 2:**
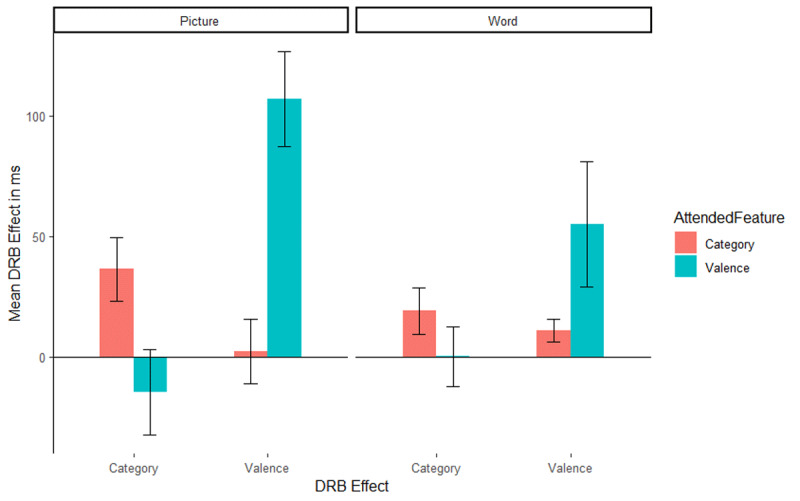
Mean distractor-response binding effects in RTs as a function of second task and stimulus material: picture stimuli of the present study (left panel) and word stimuli of the Singh et al. (2018) study (right panel). The x-axis depicts both Distractor-Response Binding effects (DRB Effect), i.e., valence-response or category-response binding effect. The y-axis depicts the mean Distractor-Response Binding effect (DRB Effect) in ms. The colour legend depicts which feature was attended. Error bars denote the standard error of the mean.

The interaction of response relation by valence relation was significant, *F*(1, 51) = 20.89, *p* < .001, η_p_^2^ = .29, indicating a significant overall valence-response binding effect. Importantly, the relevant three way interaction of attended feature by response relation by valence relation was significant, *F*(1, 51) = 19.29, *p* < .001, η_p_^2^ = .27, indicating that the valence-response binding effect differed significantly depending on whether valence was the relevant feature or not (Figure 2, left panel, the right panel presents the results from Singh et al., 2018 for reference). A post hoc comparison, *t*(45.57) = 4.43, *p* < .001, Cohen’s *d* = 1.21, indicated that the valence-response binding effect was larger when valence was the attended feature (*M* = 107 ms, *SD* = 102 ms, *t*[26] = 5.47, *p* < .001, Cohen’s *d* = 1.05) compared to when picture category was the attended feature (*M* = 2 ms *SD* = 68 ms, *t*[25] = 0.16, *p* = .875, Cohen’s *d* = 0.03). The interaction of response relation by picture category relation did not reach significance, *F*(1, 51) = 0.94, *p* = .338, η_p_^2^ = .02, indicating no evidence for an overall picture category-response binding effect. Again, the relevant three way interaction of attended feature by response relation by picture category relation was significant, *F*(1, 51) = 5.32, *p* = .025, η_p_^2^ = .09, indicating that the picture category-response binding effect differed significantly depending on whether picture category was relevant or not (Figure 2, left panel, the right panel presents the results from Singh et al., 2018, for reference). A post hoc comparison, *t*(47.46) = 2.32, *p* = .025, Cohen’s *d* = 0.63 indicated that the category-response binding effect was larger when picture category was the attended feature (*M* = 36 ms *SD* = 67 ms, *t*[25] = 2.77, *p* = .010, Cohen’s *d* = 0.54) compared to when valence was attended (*M* = –15 ms *SD* = 92 ms, *t*[26] = 0.84, *p* = .409, Cohen’s *d* = 0.16).

#### Error rates

**Figure 3:**
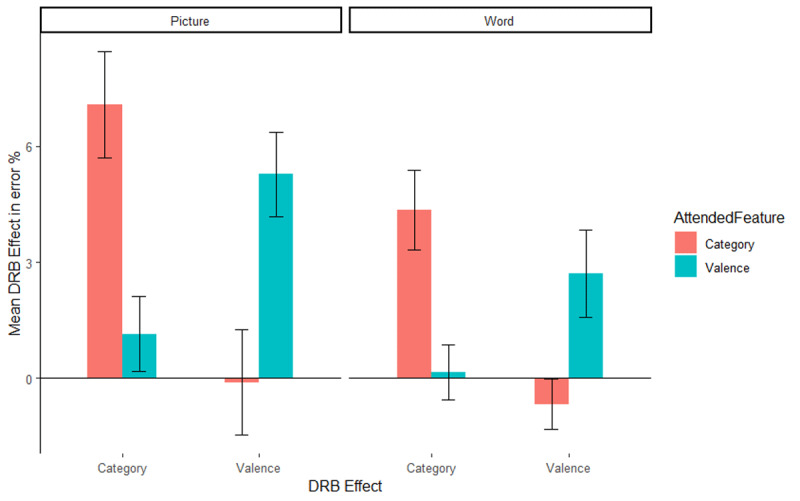
Mean distractor-response binding effects in error rates as a function of second task and stimulus material: picture stimuli of the present study (left panel) and word stimuli of the Singh et al. (2018) study (right panel). The x-axis depicts both Distractor-Response Binding effects (DRB Effect), i.e., valence-response or category-response binding effect. The y-axis depicts the mean Distractor-Response Binding effect (DRB Effect) in error percentage. The colour legend depicts which feature was attended. Error bars denote the standard error of the mean.

The same ANOVA was run on the error rates. Only trials with correct prime response were included in the error rate analysis. Mean error rates along with SD are presented in Table 3 for picture stimuli, along with the error rates from Singh et al. (2018) with word stimuli. As with the RTs, only the relevant results are presented here, the full results are presented in Table 4. The interaction of response relation by valence relation was significant, *F*(1, 51) = 8.85, *p* = .004, η_p_^2^ = .15, indicating a significant overall valence-response binding effect. Importantly, the relevant three way interaction of attended feature by response relation by valence relation was significant, *F*(1, 51) = 9.58, *p* = .003, η_p_^2^ = .16, indicating that the valence-response binding effect differed significantly depending on whether valence was the attended feature or not (Figure 3, left panel, the right panel presents the results from Singh et al., 2018 for reference). A post hoc comparison, *t*(48.29) = 3.08, *p* = .003, Cohen’s *d* = 0.85, indicated that the valence-response binding effect was larger when valence was attended (*M* = 5.27%, *SD* = 5.67%, *t*[26] = 4.83, *p* < .001, Cohen’s *d* = 0.93), compared to when picture category was attended (*M* = –0.10%, *SD* = 6.93%, *t*[25] = 0.08, *p* = .940, Cohen’s *d* = 0.01). The interaction of response relation by picture category relation was significant, *F*(1, 51) = 24.23, *p* < .001, η_p_^2^ = .32, indicating an overall picture category-response binding effect. Again, the relevant three way interaction of attended feature by response relation by picture category relation was significant, *F*(1, 51) = 12.58, *p* = .001, η_p_^2^ = .20, indicating that the picture category-response binding effect differed significantly depending on whether picture category was the attended feature or not (Figure 3, left panel, the right panel presents the results from Singh et al., 2018 for reference). A post hoc comparison, *t*(45.40) = 3.53, *p* < .001, Cohen’s *d* = 0.97, indicated that the picture category-response binding effect was larger when picture category was attended (*M* = 7.06%, *SD* = 6.98%, *t*[25] = 5.16, *p* < .001, Cohen’s *d* = 1.01) compared to when valence was attended (*M* = –1.15%, *SD* = 5.04%, *t*[26] = 1.18, *p* = .248, Cohen’s *d* = 0.23).

**Table 3:** Mean Error Rates and SD in error percent for picture stimuli and word stimuli from Singh et al. (2018).


		VR		VC	
	
		CR	CC	CR	CC
	
		MEAN (SD)	MEAN (SD)	MEAN (SD)	MEAN (SD)

Valence Attended					

RR	Picture	2.34 (3.69)	1.62 (2.48)	5.35 (4.94)	5.76 (5.83)

	Word	1.30 (2.71)	1.11 (2.02)	2.76 (4.21)	4.16 (5.69)

RC	Picture	6.39 (5.41)	5.42 (5.46)	5.03 (5.48)	3.39 (4.92)

	Word	2.91 (5.12)	2.88 (5.34)	1.96 (4.16)	2.90 (5.81)

Category Attended					

RR	Picture	2.67 (3.97)	6.39 (5.36)	2.44 (3.25)	6.41 (6.41)

	Word	1.00 (1.99)	3.93 (5.41)	1.50 (2.45)	2.75 (4.05)

RC	Picture	6.47 (6.00)	2.59 (4.08)	5.81 (5.14)	3.25 (5.37)

	Word	3.07 (2.69)	0.90 (2.13)	3.50 (4.60)	1.12 (1.83)


VR/VC = Valence repetition and valence change, CR/CC = Category repetition and category change, RR/RC = response repetition and response change.

**Table 4:** Full analysis of error rates of Experiment 1.


EFFECT	DFs	*F*	*p*	η_p_^2^

Attended Feature (A)	1, 51	0.02	.900	.00

Response Relation (R)	1, 51	1.09	.301	.02

Valence Relation (V)	1, 51	1.22	.274	.02

Category Relation (C)	1, 51	0.36	.552	.01

A × R	1, 51	0.93	.339	.02

A × V	1, 51	1.54	.220	.03

A × C	1, 51	2.26	.139	.04

R × V	1, 51	8.85	.004	.15

R × C	1, 51	24.23	<.001	.32

V × C	1, 51	0.41	.525	.01

A × R × V	1, 51	9.58	.003	.16

A × R × C	1, 51	12.58	.001	.20

A × V × C	1, 51	0.12	.734	.00

R × V × C	1, 51	0.07	.795	.00

A × R × V × C	1, 51	1.03	.316	.02


Overall, the pattern of results observed closely resembles that of Singh et al. (2018), indicating that even with picture stimuli, binding effects for valence are modulated by attention. However, in order to better compare picture and word stimuli, the present results were compared with the results obtained in Singh et al. (2018).

#### Comparison with Singh et al. (2018)

In order to test whether the binding effects were modulated by stimulus type, that is, words or images, data from the present experiment was pooled with data from Experiment 1 of Singh et al. (2018) and analysed in a 2(response relation) × 2(valence relation) × 2(stimulus category relation) × 2(attended feature) × 2(stimulus material) mixed ANOVA (ezAnova, package, Version 4.4-0) with the first three factors as repeated measures factors and the last two factors as between subject factors. Only the hypothesis relevant effects are reported, the complete analysis is reported in Table 5 for RTs and Table 6 for error rates. The binding effects are presented in Figures 2 and 3.

**Table 5:** Full results of between experiment analysis for reaction times.


EFFECT	DFs	*F*	*p*	η_p_^2^

Attended feature (A)	1, 109	2.80	.097	.03

Stimulus type (S)	1, 109	40.16	<.001	.27

Response relation (R)	1, 109	60.79	<.001	.36

Valence relation (V)	1, 109	8.15	.005	.07

Category relation (C)	1, 109	14.74	<.001	.12

A × S	1, 109	0.00	.982	.00

A × R	1, 109	6.75	.011	.06

S × R	1, 109	10.94	.001	.09

A × V	1, 109	8.53	.004	.07

S × V	1, 109	0.24	.626	.00

A × C	1, 109	3.27	.073	.03

S × C	1, 109	3.33	.071	.03

R × V	1, 109	23.60	<.001	.18

R × C	1, 109	2.28	.134	.02

V × C	1, 109	0.00	.974	.00

A × S × R	1, 109	0.23	.635	.00

A × S × V	1, 109	0.16	.690	.00

A × S × C	1, 109	0.86	.357	.01

A × R × V	1, 109	17.16	<.001	.14

S × R × V	1, 109	1.47	.229	.01

A × R × C	1, 109	6.86	.010	.06

S × R × C	1, 109	0.01	.928	.00

A × V × C	1, 109	2.94	.089	.03

S × V × C	1, 109	0.14	.705	.00

R × V × C	1, 109	0.13	.716	.00

A × S × R × V	1, 109	2.85	.094	.03

A × S × R × C	1, 109	1.44	.233	.01

A × S × V × C	1, 109	1.83	.179	.01

A × R × V × C	1, 109	0.77	.383	.01

S × R × V × C	1, 109	0.73	.395	.01

A × S × R × V × C	1, 109	0.27	.605	.00


**Table 6:** Full results of between experiment analysis for error rates.


EFFECT	DFs	*F*	*p*	η_p_^2^

Attended feature (A)	1, 109	0.04	.845	.00

Stimulus type (S)	1, 109	19.82	<.001	.15

Response relation (R)	1, 109	0.89	.346	.01

Valence relation (V)	1, 109	3.35	.070	.03

Category relation (C)	1, 109	0.00	.979	.00

A × S	1, 109	0.15	.698	.00

A × R	1, 109	1.14	.288	.01

S × R	1, 109	0.52	.472	.00

A × V	1, 109	3.84	.053	.03

S × V	1, 109	0.00	1.00	.00

A × C	1, 109	0.26	.609	.00

S × C	1, 109	1.06	.305	.01

R × V	1, 109	11.36	.001	.09

R × C	1, 109	38.38	<.001	.26

V × C	1, 109	0.53	.467	.00

A × S × R	1, 109	0.22	.638	.00

A × S × V	1, 109	0.01	.923	.00

A × S × C	1, 109	4.06	.046	.04

A × R × V	1, 109	16.72	<.001	.13

S × R × V	1, 109	2.12	.148	.02

A × R × C	1, 109	24.31	<.001	.18

S × R × C	1, 109	3.24	.074	.03

A × V × C	1, 109	0.84	.362	.01

S × V × C	1, 109	0.14	.710	.00

R × V × C	1, 109	0.00	.971	.00

A × S × R × V	1, 109	0.87	.353	.01

A × S × R × C	1, 109	0.69	.408	.01

A × S × V × C	1, 109	2.26	.135	.02

A × R × V × C	1, 109	2.33	.130	.02

S × R × V × C	1, 109	0.24	.623	.00

A × S × R × V × C	1, 109	0.06	.812	.00


The main effect of stimulus material, *F*(1, 109) = 40.16, *p* < .001, η_p_^2^ = .27, was significant, indicating that reactions times for picture (*M* = 851 ms, *SD* = 231 ms) and word (*M* = 601 ms, *SD* = 200 ms) stimuli differed significantly. The interaction of response relation and valence relation was significant, *F*(1, 109) = 23.60, *p* < .001, η_p_^2^ = .18, indicating a significant valence-response binding effect overall. This interaction was further modulated by attended feature, that is, the interaction of attended feature by response relation by valence relation was significant, *F*(1, 109) = 17.16, *p* < .001, η_p_^2^ = .14, indicating that the valence-response binding effect was modulated by the attended feature. Post-hoc comparison, *t*(73.25) = 4.04, *p* < .001, Cohen’s *d* = 0.76, indicated that the valence-response binding effect was larger when valence was attended (*M* = 79 ms, *SD* = 126 ms, *t*[56] = 4.75, *p* < .001, Cohen’s *d* = 0.63) compared to when category was attended (*M* = 7 ms, *SD* = 50 ms, *t*[55] = 1.01, *p* = .316, Cohen’s *d* = 0.14). The four-way interaction of attended feature by stimulus material by response relation by valence relation did not reach significance, *F*(1, 109) = 2.85, *p* = .094, η_p_^2^ = .03, indicating that the attentional modulation of the valence-response binding effect was not different for words and pictures. The interaction of response relation and stimulus category relation did not reach significance, *F*(1, 109) = 2.28, *p* = .134, η_p_^2^ = .02, indicating an absence of stimulus category-response binding effect overall. The interaction of attended feature by response relation by stimulus category relation was significant, *F*(1, 109) = 6.86, *p* = .010, η_p_^2^ = .06, indicating that the category-response binding effect was modulated by attended feature. Post-hoc comparison, *t*(103.84) = 2.56, *p* = .012, Cohen’s *d* = 0.48, indicated that the category-response binding effect was larger when category was attended (*M* = 27 ms, *SD* = 60 ms, *t*[55] = 3.37, *p* = .001, Cohen’s *d* = 0.45) compared to when valence was attended (*M* = –7 ms, *SD* = 80 ms, *t*[56] = 0.66, *p* = .510, Cohen’s *d* = 0.09). This three-way interaction was not further modulated by stimulus material, that is, the attended feature by stimulus type by response relation by stimulus category relation, *F*(1, 109) = 1.44, *p* = .233, η_p_^2^ = .01, did not reach significance.

The same analysis was run on the error rates. The main effect of stimulus material was significant, *F*(1, 109) = 19.82, *p* < .001, η_p_^2^ = .15, indicating that mean error rates for picture stimuli (*M* = 4.46%, *SD* = 5.17%) and word stimuli (*M* = 2.36%, *SD* = 4.10%) differed significantly. The interaction of response relation and valence relation was significant, *F*(1, 109) = 11.36, *p* = .001, η_p_^2^ = .09, indicating a significant valence-response binding effect overall. This interaction was further modulated by attended feature, that is, attended feature by response relation by valence relation interaction was significant, *F*(1, 109) = 16.72, *p* < .001, η_p_^2^ = .13, indicating that the valence-response binding effect was modulated by the attended feature. Post-hoc comparison, *t*(109.84) = 4.04, *p* < .001, Cohen’s *d* = 0.76, indicated that the valence-response binding effect was larger when valence was attended (*M* = 3.93%, *SD* = 6.04%, *t*[56] = 4.91, *p* < .001, Cohen’s *d* = 0.65) compared to when category was attended (*M* = –0.40%, *SD* = 5.35%, *t*[55] = 0.55, *p* = .574, Cohen’s *d* = 0.08). The four-way interaction of attended feature by stimulus material by response relation by valence relation, *F*(1, 109) = 0.87, *p* = .353, η_p_^2^ = .01, did not reach significance, indicating that the attentional modulation of the valence-response binding effect was not significantly different for pictures and words. The interaction of response relation and stimulus category relation was significant, *F*(1, 109) = 38.38, *p* < .001, η_p_^2^ = .26, indicating an overall category-response binding effect. Importantly, the interaction of attended feature by response relation by category relation was significant, *F*(1, 109) = 24.31, *p* < .001, η_p_^2^ = .18, indicating that the category-response binding effect was modulated by attended feature. Post-hoc comparisons, *t*(98.08) = 4.82, *p* < .001, Cohen’s *d* = 0.91, indicated that the category-response binding effect was larger when category was attended (*M* = 5.62%, *SD* = 6.38%, *t*[55] = 6.58, *p* < .001, Cohen’s *d* = 0.88) compared to when valence was attended (*M* = –0.62, *SD* = 4.45%, *t*[56] = 1.06, *p* = .295, Cohen’s *d* = 0.14). This three-way interaction was not further modulated by stimulus material, that is, attended feature by stimulus material by response relation by stimulus category relation interaction, *F*(1, 109) = 0.69, *p* = .408, η_p_^2^ = .01, that is, the attentional modulation of the category-response binding effect was not significantly different for words and pictures.

### Discussion

The aim of the present study was to further investigate the effect of attention on distractor-response binding effects and extend previous research on this question to picture stimuli. To this end, the study design and procedure of Singh et al. (2018) was replicated with the exception that instead of words, picture stimuli were used. The present results fully replicate the findings of Singh et al. (2018), indicating that valence is processed like any other stimulus feature in terms of binding and retrieval, and no evidence for any processing benefits were observed in the present study. In the present study, just like the picture category-response binding effect, the valence-response binding effect was only observed when valence was relevant in a second task. More importantly for the present purposes, the present results indicate that this was no different for picture stimuli used in the present task and the word stimuli used in Singh et al. (2018). Additionally, the size of the binding effects were not different for word and picture stimuli. These findings indicate that, at least in the context of action control, valence processing for pictures and words does not differ significantly.

While the results provide evidence only for the binding of attended distractor features, it could be argued that since either the valence or picture category feature was always relevant to the secondary task, that the match or mismatch between the prime and probe valence or category drives the observed effects.^2^ That is, it might be argued that due to the specific task demands of the experiment, participants encode the similarity between the valence/category features, for instance, whether the prime and probe are of the same valence (e.g., “positive-same”). This would result in faster responses in trials in which the prime and probe valence/category match and the response is repeated, and in trials in which the prime and probe valence/category mismatch and the response is changed. Williams (1966) observed such a response time facilitation when participants were instructed to respond to stimulus colour matches/mismatches, while the stimulus colour was irrelevant. Shorter RTs were observed for stimulus matches when the response was to be repeated, while longer RTs were observed when the colour matched, but the response changed. However, in that study, while the stimulus colour could change independently of the response relation and match or mismatch the previous colour, it was still relevant to the RT task, in order to detect a match/mismatch and carry out the appropriate response. In the present study, the valence/type was only relevant to the secondary task (where no binding effects were measured) and not relevant to the colour classification task in which binding effects were measured. Nevertheless, it could be argued that the findings are the result of the specific task used and not necessarily due to binding (and retrieval) per se. In order to try to tackle this issue, therefore, a second study was run in which participants only had to classify the valence or picture category of the prime stimulus.

## Experiment 2

### Participants

The sample size calculation was the same as in Experiment 1. A total of 60 participants (44 female) were tested. The median age of the participants was 24.5 years (range 19–44). One participant reported doing the secondary task incorrectly by always classifying the probe stimulus instead of the prime stimulus, their data was replaced with a new participant. Two participants were excluded as due to being outliers in error rates, leading to a final sample of 58 participants. The results of the entire sample, including the outliers, is reported in **Appendix C**. The experiment was pre-registered (https://aspredicted.org/fw6b-tzkc.pdf).

### Design, materials, and procedure

The design, materials, and procedure of Experiment 2 were exactly the same as in Experiment 1, with the only exception that participants were only asked to classify the valence or picture category of the prime image on 75% of the trials, but never of the probe image. This ensures that the valence or picture category of the probe image, is always irrelevant to both tasks.

### Results

#### Reaction times

**Figure 4:**
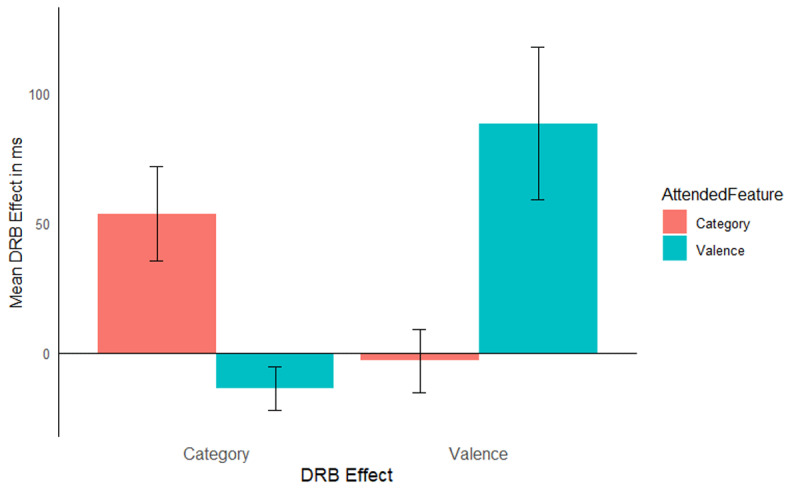
Mean distractor-response binding effects in RTs as a function of second task for Experiment 2. The x-axis depicts both Distractor-Response Binding effects (DRB Effect), i.e., valence-response or category-response binding effect. The y-axis depicts the mean Distractor-Response Binding effect (DRB Effect) in ms. The colour legend depicts which feature was attended. Error bars denote the standard error of the mean.

The same analysis was run as in Experiment 1, mean RTs are reported in Table 7. As in Experiment 1, only the hypothesis relevant results are reported here, the full analysis results are presented in Table 8. The same exclusion criteria were used as in Experiment 1, leading to an exclusion of 14.38% of trials (5.6% RT outliers, 0.03% shorter than 200 ms, and 8.8% due to errors in either the prime response, probe response, or both). The interaction of response relation by valence relation was significant, *F*(1, 56) = 7.24, *p* = .009, η_p_^2^ = .11, indicating a significant valence-response binding effect overall. Importantly, this interaction was further modulated by attended feature, *F*(1, 56) = 8.26, *p* = .006, η_p_^2^ = .13, indicating that the valence-response binding effect was modulated by attention (Figure 4). A post hoc comparison, *t*(37.35) = 2.88, *p* = .007, Cohen’s *d* = 0.75, indicated that the valence-response binding effect was larger when prime valence was attended (*M* = 88 ms, *SD* = 158 ms, *t*[28] = 3.01, *p* = .005, Cohen’s *d* = 0.56) compared to when prime picture category was attended (*M* = –3 ms, *SD* = 65 ms, *t*[28] = 0.24, *p* = .812, Cohen’s *d* = 0.04). The response relation by picture category relation interaction missed significance, *F*(1, 56) = 3.93, *p* = .052, η_p_^2^ = .07, however, the three way interaction of attended feature by response relation by picture category relation was significant, *F*(1, 56) = 11.28, *p* = .001, η_p_^2^ = .17. A post hoc comparison, *t*(39.57) = 3.36, *p* = .001, Cohen’s *d* = 0.88, indicated a larger category-response binding effect when prime picture category was relevant (*M* = 53 ms; *SD* = 98 ms, *t*[28] = 2.95, *p* = .006, Cohen’s *d* = 0.55) compared to when prime valence was relevant (*M* = –13 ms; *SD* = 45 ms, *t*[28] = 1.63, *p* = .114, Cohen’s *d* = 0.30).

**Table 7:** Mean RTs and SD in ms for each condition in Experiment 2.


	VR		VC	
	
	CR	CC	CR	CC
	
	MEAN (SD)	MEAN (SD)	MEAN (SD)	MEAN (SD)

Valence Attended				

RR	722 (212)	714 (189)	785 (267)	788 (307)

RC	785 (285)	792 (269)	761 (232)	776 (225)

Category Attended				

RR	781 (235)	824 (269)	769 (218)	814 (257)

RC	820 (235)	805 (225)	807 (233)	803 (228)


VR/VC = Valence repetition and valence change, CR/CC = Category repetition and category change, RR/RC = response repetition and response change.

**Table 8:** Full results of analysis of RTs for Experiment 2.


EFFECT	DFs	*F*	*p*	η_p_^2^

Attended Feature (A)	1, 56	0.35	.556	.01

Response Relation (R)	1, 56	10.44	.002	.16

Valence Relation (V)	1, 56	1.50	.226	.03

Category Relation (C)	1, 56	6.46	.014	.10

A × R	1, 56	1.60	.211	.03

A × V	1, 56	7.51	.008	.12

A × C	1, 56	2.34	.132	.04

R × V	1, 56	7.24	.009	.11

R × C	1, 56	3.93	.052	.07

V × C	1, 56	0.64	.427	.01

A × R × V	1, 56	8.26	.006	.13

A × R × C	1, 56	11.28	.001	.17

A × V × C	1, 56	0.02	.829	.00

R × V × C	1, 56	0.06	.813	.00

A × R × V × C	1, 56	0.19	.662	.00


#### Error rates

**Figure 5:**
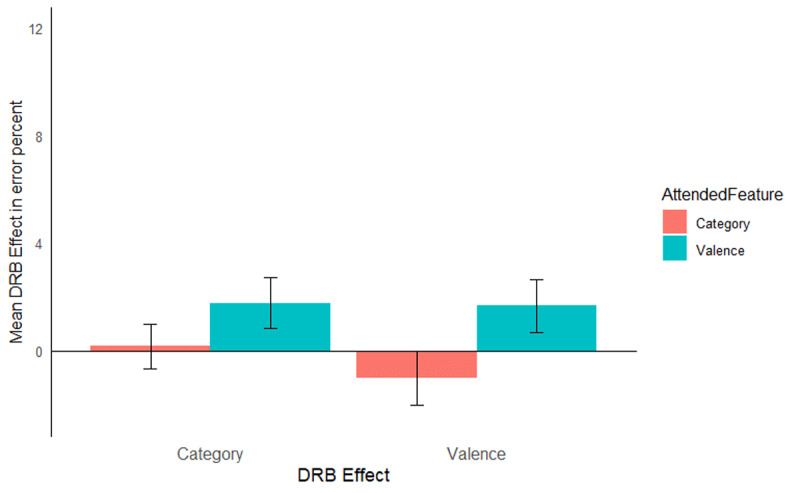
Mean distractor-response binding effects in error rates as a function of second task for Experiment 2. The x-axis depicts both Distractor-Response Binding effects (DRB Effect), i.e., valence-response or category-response binding effect. The y-axis depicts the mean Distractor-Response Binding effect (DRB Effect) in error percentage. The colour legend depicts which feature was attended. Error bars denote the standard error of the mean.

The same analysis was run on the error rates, mean error rates are presented in Table 9. Only relevant effects are reported here, the full results are reported in Table 10. The interaction of response relation and valence relation did not reach significance, *F*(1, 56) = 0.21, *p* = .649, η_p_^2^ = .00, and was not further modulated by attended feature, *F*(1, 56) = 3.63, *p* = .062, η_p_^2^ = .06. Similarly, the response relation and picture type interaction was not significant, *F*(1, 56) = 0.26, *p* = .612, η_p_^2^ = .00, and was not further modulated by attended feature, *F*(1, 56) = 1.67, *p* = .201, η_p_^2^ = .03 (Figure 5).

**Table 9:** Mean error rates and SD in error percent for Experiment 2.


	VR		VC	
	
	CR	CC	CR	CC

	MEAN (SD)	MEAN (SD)	MEAN (SD)	MEAN (SD)

Valence Attended				

RR	3.77 (4.04)	4.20 (4.20)	3.88 (4.55)	6.36 (5.63)

RC	4.85 (5.32)	4.85 (4.98)	4.63 (4.84)	3.99 (5.14)

Category Attended				

RR	4.09 (4.66)	4.20 (4.60)	2.91 (3.23)	4.74 (4.39)

RC	3.56 (5.06)	4.20 (4.60)	4.09 (4.88)	5.06 (6.05)


VR/VC = Valence repetition and valence change, CR/CC = category repetition and category change, RR/RC = response repetition and response change.

**Table 10:** Full results of the analysis of error rates for Experiment 2.


EFFECT	DFs	*F*	*p*	η_p_^2^

Attended Feature (A)	1, 56	0.23	.631	.00

Response Relation (R)	1, 56	0.17	.681	.00

Valence Relation (V)	1, 56	0.70	.405	.01

Category Relation (C)	1, 56	5.28	.025	.09

A × R	1, 56	0.11	.743	.00

A × V	1, 56	0.03	.853	.00

A × C	1, 56	0.26	.612	.00

R × V	1, 56	0.21	.649	.00

R × C	1, 56	2.41	.127	.04

V × C	1, 56	2.15	.148	.04

A × R × V	1, 56	3.63	.062	.06

A × R × C	1, 56	1.67	.201	.03

A × V × C	1, 56	0.08	.784	.00

R × V × C	1, 56	2.91	.093	.05

A × R × V × C	1, 56	0.29	.592	.01


### Discussion

The aim of Experiment 2 was to exclude the alternative explanation that encoding the prime-probe valence or category matches drives the observed binding effect. To this end, participants were instructed to only report the prime valence or category in Experiment 2, thus rendering the probe valence or category completely irrelevant to both tasks, while still attending to the valence/category in the prime. The results of Experiment 2 closely mirror those of Experiment 1. Even though probe valence was completely irrelevant to both tasks, significant valence-response and category-response binding effects were only observed when that feature was attended. That is, larger valence-response binding effects when prime valence was relevant compared to when prime picture category was relevant and larger category-response binding effects when prime picture category was relevant compared to when prime valence was relevant. These results thus provide evidence that present findings are indeed due to binding and retrieval effects rather than to a specific methodological or procedural feature of the task itself.

## General Discussion

The aim of the present study was to determine whether valence-response binding effects are susceptible to attentional allocation. While previous studies indeed indicated that this might be the case (e.g., Singh et al., 2018) binding effects were only tested with affective words. Given that affective processing has sometimes been observed to be independent of attentional demands (e.g., Kissler et al., 2009) and that differences in valence processing for pictures and words has been observed (e.g., Spruyt et al., 2002), the present study tested whether valence-response binding effects with picture stimuli would emerge even when attention was directed towards a different stimulus feature. In two studies, participants responded to the colour of the border of a picture. The valence and picture category of the stimulus was irrelevant to the colour classification task, but relevant to a secondary task. In Experiment 1 the valence or picture category of both the prime and probe stimuli were relevant to the secondary task, while in Experiment 2 only the prime stimulus valence or picture category was relevant. In both studies binding effects were only observed for the feature that was relevant to the secondary task. A between experiment analysis including the data of Experiment 1 and Singh et al. (2018) indicated that while reaction times were faster and less error prone for word stimuli compared to picture stimuli, there was no difference in the strength of the binding effects, and attentional allocation influenced the binding effects in the same manner for both types of stimuli. The longer reaction times for picture stimuli relative to word stimuli might be a function of increased working memory load and/or increased complexity of the picture stimuli relative to the word stimuli. Although previous findings with respect to increased load indicate that binding effects should disappear under increased load (e.g., Singh & Schubert, 2021), the possibility that increased load led to longer reaction times for the picture stimuli cannot be ruled out completely in the present study.

One issue of general relevance to the discussion is the use of the tem *distractor* in the present context. In the present context features that were not relevant to the primary speeded colour classification task were both termed distractors. These features were only relevant to a secondary task carried out in parallel, for which no binding effects were measured, that is, the valence or category classification task. The central idea of the secondary task here was to direct attention to one or the other feature, without making them in any way response relevant in the primary task, so that binding effects for each of the features could be measured without them ever being response relevant. This by no means implies that these features are generally irrelevant/distractors. Indeed the intentional weighting account (Memelink & Hommel, 2013) postulates that the cognitive system weights features depending on their relevance to action goals, with features that are more relevant to the action goals receiving higher weights than those that are not relevant, and this in turn makes them more likely to be integrated in event files. Thus, in the present context, the attended feature would receive higher weights since it is relevant to the secondary task, which is carried out in parallel. However, importantly, it is never relevant to the primary task. Thus, it is a *distractor* in the context of the primary task.

### Binding effects in picture vs. word stimuli

The present results replicate the findings of Singh et al. (2018) and further generalise them to a different stimulus material, namely, picture stimuli. Although it has been argued that picture stimuli and word stimuli are processed differently (e.g., Beall & Herbert, 2008; Flaisch et al., 2015; Rellecke et al., 2011) and that affective priming effects are more reliable with picture stimuli (Spruyt, et al., 2002), the present results indicate that the findings of Singh et al. (2018) were not driven by stimulus material. Even with picture stimuli, which have been suggested to have privileged access to affective information (De Houwer & Hermans, 1994), have a processing speed advantage over words (Schacht & Sommer, 2009) or hypothesised to be less easily supressed or more easily amplified than word stimuli (Kunde et al., 2012), valence-response binding effects only emerged when valence was relevant to a secondary task, but not when picture category was relevant. Similarly, category-response binding effects were only observed when picture category was relevant, but not when valence was relevant. Thus, although emotional picture and word stimuli might be processed differently, that is, at different speed (Schacht & Sommer, 2009, however see also Bruno et al., 2020) or in different regions or networks (e.g., Feng et al., 2021; Flaisch et al., 2015) they both produce valence-response binding effects which are similarly subject to attentional modulations. The present findings regarding binding effects specifically, are at odds with studies observing differences between picture and word stimulus driven effects, for example, larger interference effects for affective pictures relative to words (e.g., Beall & Herbert, 2008; De Houwer & Hermans, 1994), different spatial attentional modulations via affective pictures relative to words (Sutton & Lutz, 2019), or more reliable affective priming effects using pictures versus words (Spruyt et al., 2002). The present results also indicate that for both stimulus types, valence-response binding effects occur only when attention is directed towards the affective feature. At the same time, the results indicated that, participants were faster and made fewer mistakes with word stimuli compared to picture stimuli (see also Sutton & Lutz, 2019), presumably because pictures are more attention grabbing than words. Thus, while stimulus material may indeed have an effect on responding, and valence may well be processed differently for picture and word stimuli in some contexts, binding effects for picture and word stimuli are similarly modulated by feature-based attention manipulations.

### Automaticity of valence processing

The present results are in line with a number of other studies indicating that valence processing is not wholly automatic and is indeed subject to attentional demands (e.g., De Houwer & Randell, 2002; Doallo et al., 2006; Gupta et al., 2016; Pessoa et al., 2002a, 2002b; Schupp et al., 2006; Spruyt et al., 2009; Wiens & Syrjänen, 2013). In a number of priming studies, no influence of valence or affect has been observed unless valence was task relevant, that is, affective priming effects are only observed in tasks in which participants must classify the valence of the stimulus, but not when any other semantic or lexical category must be classified (e.g., De Houwer et al., 2002; Spruyt et al., 2009). For instance, De Houwer et al. (2002) did not observe affective priming effects in their Experiment 1, in which participants were instructed to classify whether the target was a person or an animal. In Experiment 2, however, when participants were instructed to classify the valence of the stimuli, affective priming effects were observed. That is, affective priming effects were only observed when participants were instructed to classify the valence of the stimuli. Spruyt et al. (2009) instructed participants to classify the stimuli as positive or negative on either 25% or 75% of the trials. In the rest of the trials, participants were instructed to read the stimulus out aloud. They only observed affective priming effects in the condition in which participants classified the valence of the stimuli in 75% of the trials. Such findings indicate that valence processing is subject to attentional allocation. The present results further underscore this conclusion that valence processing is not independent of attentional resources, rather, attention – in the present case feature-based attention – is required for valence processing. Additionally, studies implementing different tasks also observe effects of valence only when enough resources are available. For instance, Doallo et al. (2006) had participants classify the size of two sequentially presented stimuli. Additionally, they also presented positive or negative stimuli peripherally between the two sequential stimuli. They only observed a difference in the N1-P2 ERP component for neutral and negative stimuli under the easy task conditions condition, but no significant difference under difficult task condition. Similarly, Gupta et al. (2016) also observed decreased processing of emotional distractors under high perceptual load conditions, but not under low load conditions.

However, while there is evidence against the attention independent processing of valence, some studies do observe exactly this. Fischer and Schubert (2008) observed that valence information was able to bypass the bottleneck stage – so called due to resource constraints at response processing stages – however, this is not specific to valence. Oriet et al., (2005) showed that even other types of categorical information like number categories can bypass the bottleneck stage. Other studies, for instance, Pecchinenda and Heil (2007) observed interference effects via affective distractors were similar under conditions of high working memory load and low working memory load. Kissler et al. (2009) observed no differences in the early posterior negativity and late positive complex ERP components in conditions in which participants were asked to attend to a lexical feature of the stimulus word compared to when they received no such instruction. Kissler et al. (2009) suggested that attention effects on valence processing can be independent of attentional demands via other tasks, if both valence processing and any other task processing rely on different neural structures and different processing latencies. One finding especially relevant to the present purposes was reported in Schöpper, Jerusalem, et al. (2023). In their study, participants carried out a discrimination task (the letter ‘W’ vs. ‘M’). Participants were instructed to respond via touching the respective response field on a touchpad. During the prime display, the response fields were overlaid with images of either spiders or fruits. Schöpper, Jerusalem, et al. (2023) observed that pressing the field with a spider led to shorter response times in response repetition trials relative to pressing the field with a fruit, thus indicating that response repetitions are slower for aversive stimuli. This finding was interpreted as evidence for valence-response binding effects. Thus, even though the valence of the fruits/spider stimuli were not relevant to the task, they were still bound to the response. While the present findings somewhat contradict the findings of Schöpper, Jerusalem, et al. (2023), methodological differences between the studies must be considered. Firstly, in the present study, two irrelevant features were presented, and, secondly, attention was explicitly directed to either one of them via the secondary task. Third, the current study involved memorization and reporting of an attended feature, likely demanding additional cognitive resources. In Schöpper, Jerusalem, et al. (2023) no attentional manipulation was included, no further distractors were presented, and there was no memory task used. Thus, irrelevant valence features can be bound to responses, however, this is only the case when either no other competing distractor features are present and/or enough attentional resources are available.

One potential limitation of the present study is the task difficulty. Subjective reports at debriefing indicated that participants experienced the colour classification task as very difficult. This is reflected in the proportion of excluded trials. In the present study 15.24% of the trials were excluded from the RT analysis, while in Singh et al., (2018) only 11.45% of the trials were excluded. Another important consideration is that the present study included a mix of images of sceneries, animals and humans but no images of faces in isolation. It is well known that faces are processed differently to other visual stimuli, and, additionally, faces are socially relevant stimuli for humans. Therefore, it is possible that affective information is extracted differently from faces; future studies might examine whether valence-binding effects with face stimuli are less susceptible to attentional modulations. First evidence that facial expressions are integrated with actions has already been observed (e.g., Coll & Grandjean, 2016; Coll et al., 2019). Additionally, there was no differentiation between high and low valence stimuli. For instance, Fazio et al. (1986) observed stronger priming effects for stimuli with a stronger attitude associations. Future studies might test the effects of strong vs. weak stimulus valence separately.

In conclusion, the present findings replicate the previous findings of Singh et al. (2018) and generalise them to picture stimuli. Secondly, the present results underscore the relevance of attentional allocation for stimulus-response binding effects. Finally, the present results also provide further evidence that valence is processed just like any other stimulus feature and is subject to attentional modulations in the context of action control.

## Data Accessibility Statement

The data for the study can be found here: https://doi.org/10.23668/psycharchives.16034.

Data for Singh et al. 2018 can be found here: http://dx.doi.org/10.23668/psycharchives.14406.

## Additional File

The additional file for this article can be found as follows:

10.5334/joc.432.s1 Appendices. Appendix A to C.

## Footnotes

The analysis with the full sample, that is, including outliers, is included in Appendix A.

We would like to thank one of the reviewers, Prof. Dr. Thomas Kleinsorge, for suggesting this alternative.
