# Supplementary material for: I am Once Again Asking for Your Attention: A Replication of Feature-Based Attention Modulations of Binding Effects with Picture Stimuli
Source: J Cogn. 2025 Feb 5;8(1):22. doi: 10.5334/joc.432 (PMC11804182; doi:10.5334/joc.432)
Supplement: Appendices. — Appendix A to C. [file joc-8-1-432-s1.pdf]

Appendix A.

Full results of Experiment 1 reaction times without excluding any outliers. One participant was excluded as they did not have any correct responses in at least one condition.

**Effect**	**DFs**	***F***	***p***	η_p^2
Attended Feature (A)	1, 57	0.81	.371	.01
Response Relation (R)	1, 57	6.64	.013	.10
Valence Relation (V)	1, 57	1.58	.215	.03
Category Relation (C)	1, 57	9.12	.004	.14
A x R	1, 57	2.53	.117	.04
A x V	1, 57	2.62	.111	.04
A x C	1, 57	1.79	.186	.03
R x V	1, 57	18.25	< .001	.24
R x C	1, 57	1.32	.256	.02
V x C	1, 57	0.11	.740	.00
A x R x V	1, 57	17.80	< .001	.24
A x R x C	1, 57	4.11	.047	.07
A x V x C	1, 57	1.69	.199	.03
R x V x C	1, 57	0.00	.994	.00
A x R x V x C	1, 57	0.01	.937	.00

Full results of Experiment 1 error rates without excluding any outliers. One participant was excluded as they did not have any correct responses in at least one condition.

**Effect**	**DFs**	***F***	***p***	η_p^2
Attended Feature (A)	1, 57	0.02	.880	.00
Response Relation (R)	1, 57	1.82	.183	.03
Valence Relation (V)	1, 57	0.68	.414	.01
Category Relation (C)	1, 57	0.03	.870	.00
A x R	1, 57	3.40	.070	.06
A x V	1, 57	0.24	.628	.00
A x C	1, 57	2.47	.122	.04
R x V	1, 57	10.55	.002	.16
R x C	1, 57	19.04	< .001	.25
V x C	1, 57	0.06	.814	.00
A x R x V	1, 57	7.16	.010	.11
A x R x C	1, 57	15.73	< .001	.22
A x V x C	1, 57	0.47	.497	.01
R x V x C	1, 57	0.11	.744	.00
A x R x V x C	1, 57	1.07	.306	.02

Appendix B.

Image IDs along with valence rating and category.

ID	Valence (SD)	Affective Category	Type Category	ID	Valence (SD)	Affective Category	Type Category
IAPS							
1440	8.19 (1.53)	Non-Aversive	Animate	7330	7.69 (1.84)	Non-Aversive	Inanimate
1441	7.79 (1.28)	Non-Aversive	Animate	7350	7.10 (1.98)	Non-Aversive	Inanimate
1463	7.45 (1.76)	Non-Aversive	Animate	7360	3.59 (1.95)	Aversive	Inanimate
1620	7.73 (1.56)	Non-Aversive	Animate	7487	4.92 (1.57)	Aversive	Inanimate
1750	8.28 (1.07)	Non-Aversive	Animate	7508	7.02 (1.46)	Non-Aversive	Inanimate
2154	8.03 (1.13)	Non-Aversive	Animate	8185	7.57 (1.52)	Non-Aversive	Animate
2209	7.64 (1.46)	Non-Aversive	Animate	8420	7.76 (1.55)	Non-Aversive	Animate
2216	7.57 (1.31)	Non-Aversive	Animate	8470	7.74 (1.53)	Non-Aversive	Animate
2260	8.06 (1.42)	Non-Aversive	Animate	8501	7.91 (1.66)	Non-Aversive	Inanimate
2455	2.96 (1.79)	Aversive	Animate	5810	7.32 (1.72)	Non-Aversive	Inanimate
2683	2.62 (1.78)	Aversive	Animate	9830	2.54 (1.75)	Aversive	Inanimate
2710	2.52 (1.69)	Aversive	Animate	9901	2.27 (1.25)	Aversive	Inanimate
4613	5.34 (1.77)	Aversive	Inanimate	9903	2.36 (1.35)	Aversive	Inanimate
5450	7.01 (1.60)	Non-Aversive	Inanimate	9911	2.30 (1.37)	Aversive	Inanimate
5480	7.53 (1.63)	Non-Aversive	Inanimate				
5551	7.31 (1.63)	Non-Aversive	Inanimate	GAPED			
5600	7.57 (1.48)	Non-Aversive	Inanimate	A026	31.85 (27.67)	Aversive	Animate
5760	8.05 (1.23)	Non-Aversive	Inanimate	A027	14.33 (16.09)	Aversive	Animate
5920	5.16 (1.92)	Aversive	Inanimate	A033	11.41 (13.99)	Aversive	Animate
5950	5.99 (2.07)	Aversive	Inanimate	A056	17.08 (14.87)	Aversive	Animate
5970	4.14 (1.77)	Aversive	Inanimate	A119	18.39 (24.10)	Aversive	Animate
6230	2.37 (1.57)	Aversive	Inanimate	A131	13.62 (14.62)	Aversive	Animate
7034	4.95 (0.87)	Aversive	Inanimate	H104	13.38 (11.93)	Aversive	Animate
7230	7.38 (1.65)	Non-Aversive	Inanimate	H105	5.23 (7.32)	Aversive	Animate
7280	7.20 (1.80)	Non-Aversive	Inanimate	H126	21.03 (16.92)	Aversive	Animate

Appendix C.

Full results of Experiment 2 reaction times without excluding any outliers.

**Effect**	**DFs**	***F***	***p***	η_p^2
Attended Feature (A)	1, 58	0.15	.705	.00
Response Relation (R)	1, 58	11.38	.001	.16
Valence Relation (V)	1, 58	2.18	.145	.04
Category Relation (C)	1, 58	5.90	.018	.09
A x R	1, 58	1.69	.198	.03
A x V	1, 58	9.10	.004	.14
A x C	1, 58	2.81	.099	.05
R x V	1, 58	6.59	.013	.10
R x C	1, 58	4.94	.030	.08
V x C	1, 58	0.09	.765	.00
A x R x V	1, 58	7.69	.007	.12
A x R x C	1, 58	7.84	.007	.12
A x V x C	1, 58	0.04	.842	.00
R x V x C	1, 58	0.00	.968	.00
A x R x V x C	1, 58	0.17	.684	.00

Full results of Experiment 2 error rates without excluding any outliers.

**Effect**	**DFs**	***F***	***p***	η_p^2
Attended Feature (A)	1, 58	0.58	.448	.01
Response Relation (R)	1, 58	0.00	1.00	0.00
Valence Relation (V)	1, 58	0.16	.691	.00
Category Relation (C)	1, 58	3.85	.054	.06
A x R	1, 58	0.66	.420	.01
A x V	1, 58	0.23	.634	.00
A x C	1, 58	0.54	.465	.01
R x V	1, 58	0.07	.792	.00
R x C	1, 58	2.78	.101	.05
V x C	1, 58	2.52	.118	.04
A x R x V	1, 58	2.32	.133	.04
A x R x C	1, 58	3.06	.086	.05
A x V x C	1, 58	0.01	.930	.00
R x V x C	1, 58	2.27	.137	.04
A x R x V x C	1, 58	0.28	.597	.00
